# Longevity, tumor, and physical vitality in rats consuming ginsenoside Rg1

**DOI:** 10.1016/j.jgr.2021.04.006

**Published:** 2021-04-22

**Authors:** Chao-Chieh Hsieh, Chiung-Yun Chang, Tania Xu Yar Lee, Jinfu Wu, Suchada Saovieng, Yu-Wen Hsieh, Maijian Zhu, Chih-Yang Huang, Chia-Hua Kuo

**Affiliations:** a Laboratory of Exercise Biochemistry, University of Taipei, Taipei, Taiwan; b Laboratory of Regenerative Medicine in Sports Science, School of Physical Education & Sports Science, South China Normal University, Guangzhou, China; c College of Sports Science & Technology, Mahidol University, Thailand; d Cardiovascular and Mitochondrial Related Disease Research Center, Hualien Tzu Chi Hospital, Buddhist Tzu Chi Medical Foundation, Hualien, Taiwan; e Center of General Education, Buddhist Tzu Chi Medical Foundation, Tzu Chi University of Science and Technology, Hualien, Taiwan; f Department of Medical Research, China Medical University Hospital, China Medical University, Taichung, Taiwan; g Graduate Institute of Biomedical Sciences, China Medical University, Taichung, Taiwan; h Department of Medical Laboratory Science and Biotechnology, Asia University, Taichung, Taiwan

**Keywords:** Ginseng, Inflammation, TNF-α, IL-10, Oxidized LDL, Obesity, Muscle mass, Tumor necrosis factor-α, (TNF-α), Interleukin-10, (IL-10), Oxidized low density lipoprotein, (oxidized LDL), Institutional Animal Care and Use Committee, (IACUC), High-fructose corn syrup, (HFCS), Two-way analysis of variance, (ANOVA), Standard error, (SE), Dual-energy x-ray absorptiometry, (DEXA)

## Abstract

**Background:**

Effects of the major ginsenoside Rg1 on mammalian longevity and physical vitality are rarely reported.

**Purpose:**

To examine longevity, tumor, and spontaneous locomotor activity in rats consuming Rg1.

**Methods:**

A total of 138 Wistar rats were randomized into 2 groups: control (N = 69) and Rg1 (N = 69). Rg1 (0.1 mg/kg per day) were orally supplemented from 6 months of age until natural death. Spontaneous mobility was measured by video-tracking together with body composition (dual energy x-ray absorptiometry) and inflammation markers at 5, 14, 21, and 28 months of age.

**Results:**

No significant differences in longevity (control: 706 days; Rg1: 651 days, *p* = 0.77) and tumor incidence (control: 19%; Rg1: 12%, *p* = 0.24) were observed between the two groups. Movement distance in the control group declined significantly by ∼60% at 21 months of age, together with decreased TNF-α (*p* = 0.01) and increased IL-10 (*p* = 0.02). However, the movement distance in the Rg1 group was maintained ∼50% above the control groups (*p* = 0.01) at 21 months of age with greater magnitudes of TNF-α decreases and IL-10 increases. Glucose, insulin, and body composition (bone, muscle and fat percentages) were similar for both groups during the entire observation period.

**Conclusion:**

The results of the study suggest a delay age-dependent decline in physical vitality during late life by lifelong Rg1 consumption. This improvement is associated with inflammatory modulation. Significant effects of Rg1 on longevity and tumorigenesis were not observed.

## Graphical abstract

**Figure:**
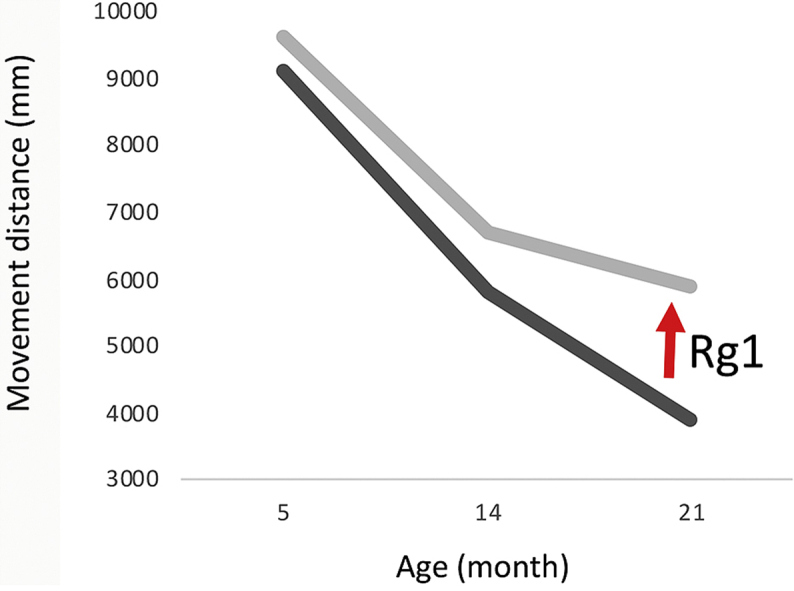

## 1 Introduction

*Panax ginseng* has traditionally been thought to have life-prolonging and vitality-enhancing properties. However, scientific evidence has not been well-established in mammals [1,2]. Such data is also a key reference of toxicity for long-term ginseng use. *In vivo* studies using ginseng as testing materials suffer inconsistency in major ginsenoside profile due to seasonal changes and geographical variations, leading to uncertain efficacy [3,4]. The major ginsenoside Rg1 and Rb1 show opposing metabolic action [5,6]. Therefore, it is difficult to answer the question of whether ginseng can improve longevity and physical vitality unless its ginsenoside component is well-controlled.

Among the major ginsenoside components in panax ginseng, Rg1 has been reported to reverse loss of spontaneous physical activity of young mice under neuronal damaged condition [7] and increases senescence cell clearance in human muscle after exercise [8]. However, most of the Rg1 supplementation studies in mammals are short-term (<1 month) and were conducted in animals at young age [9,10]. Physical vitality declines during late life and is closely associated with survival time in both animals and humans [11]. It remains unknown whether lifelong Rg1 supplementation can reverse age-dependent decreases in aging mammal’s physical vitality.

Anti-inflammatory action of Rg1 has been previously reported [12], implicating that the beneficial effect of Rg1 supplementation is mediated by immunomodulatory action. An increase in systemic inflammation during advancing age is currently recognized as a common predictor for tumorigenesis and metabolic problems [13]. According to a cross-sectional study, plasma IL-10 and M2 macrophage in tissues of old animals are greater than young animals [14], suggesting a compensatory mechanism to maintain the increasingly growing body size by strengthening the regenerative phase of inflammation [15]. During inflammation, TNF-α increases during the early phagocytic phase, whereas IL-10 increases during the late regenerative phase before resolution of the inflammation [16]. Effect of Rg1 supplementation on trajectories of plasma TNF-α and IL-10 during aging have not previously been reported. To determine the effect of Rg1 on longevity and physical vitality, we measured survival time and spontaneous physical activity in rats consuming ginsenoside Rg1 from 6 months of age until natural death. Inflammatory markers, glucose, insulin, oxidized low density lipoprotein (oxidized LDL), tumor incidence and body fat were assessed at 5 (before treatment), 14, 21, 28 months of age.

## 2 Materials and methods

### 2.1 Animals

This study was approved by the Animal Care and Use Committee at the University of Taipei (approval number UT104004) and conformed to the ethical regulation of the Institutional Animal Care and Use Committee (IACUC) and in accordance with the Law of Taiwan in animal protection. Wistar rats were purchased from BioLASCO Taiwan Corporation (Yi-Lan, Taiwan) at 4 weeks with known date of birth. They were housed in the Animal Center of the University of Taipei (Tianmu Campus, Taipei, Taiwan). Animals were maintained in a thermostatic facility with the controlled temperature of 22°C, relative humidity of ∼50%, 12/12 h light/dark cycle. Rats (two per cage) were provided with standard laboratory chow LabDiet 5001 (LabDiet, Missouri, USA) and tap water *ad libitum*. Institutional regulation for enforced euthanasia (IACUC decisions after a judge by a veterinarian) are tumor size greater than 5 cm with unhealed wound, weight loss > 20%, and decreased physical activity. A total of 13 rats in the control group (first-last: 424-907 d) and 14 rats in the Rg1 group (first-last: 417-907 d) were euthanatized. Euthanatized rats were excluded for longevity analysis (Control: N = 13; Rg1: N = 14) and were included in tumor incidence calculation.

### 2.2 Study design

This study used 138 rats and ranked into 69 weight levels at 1 month of age. They were randomized into the control group (F = 34; M = 35) and the Rg1 group (F = 34, M = 35) for each weight level to minimize potential effect of different growth rate on outcome variables. Rats in the control group received 11% high-fructose corn syrup (HFCS) drink, whereas Rg1 group received 11% HFCS drink containing 0.1 mg/kg of Rg1 from 6 months of age until natural death. HFCS drinks were provided when rats reached 3 months of age before Rg1 treatment. Rg1 concentration in the drink was adjusted weekly according to consumed volume of the drink to maintain the targeted dose of Rg1 at 0.1 mg/kg/day. The dosages at both 0.1 mg/kg and 0.01 mg/kg have been previously found to minimize glucose response in oral glucose tolerance test in rats for short-term use [5]. In human study, 0.08 mg/kg has been found to improve exercise-induced cellular senescence in exercised muscle without noticeable side effect [17]. Ginsenoside Rg1 was obtained from NuLiv Science, Inc. (Brea, CA, USA). Rg1 was dissolved using N-methyl-2-pyrrolidone (no-observed effect level of 169 mg/kg/day) in HFCS drink [18]. HFCS drinks for the control group also contained the same amount of N-methyl-2-pyrrolidone.

### 2.3 Metabolic measures

After a 12-h fasting, blood glucose of rats was measured immediately after sample collection from tail using Accu-chek® performa system (Roche Diagnostics, Indiana, USA). Blood sample was collected in EDTA-contained tubes. Plasma was obtained after centrifugation at 4°C, 3000 rpm for 10 min. Other plasma measures were analyzed using an enzyme-linked immunosorbent assay (ELISA) reader (Tecan GENios, A-5082, Austria) with commercial kits. Plasma insulin was measured using ELISA kit from Mercodia Inc. (#10-1250-01) (Mercodia AB, Uppsala, Sweden). Oxidized LDL was measured using ELISA kits from CusaBio technology LLC (#CSB-E07932r) (Houston, Texas, USA). TNF-α and IL-10 were measured using LEGEND MAX™ Rat TNF-α kit (#438207) from Biolegend Inc (San Diego, California, USA) and Rat IL-10 Quantikine ELISA Kit (#R1000) from R&D Systems (Minneapolis, Minnesota, USA), respectively.

### 2.4 Spontaneous physical activity

Physical vitality was monitored using Locoscan system (Clever Sys, VA, USA). Rats were transferred from their housing cage to the testing cage (40 cm^3^ black plastic cage) before activity assessment. One week prior to the assessment, rats were placed in the testing cage for 10 min daily for acclimation. Spontaneous activity was performed in a quiet, dark, and cleaned environment. Rats were placed into the center of testing cage and free to move. A digital infrared camera was mounted above the center of testing cage to record video on their physical activity for 7 min. The middle 5-min recording periods were used from analysis to exclude possible disturbances related to human access to dark room. Rat movement was detected based on video-tracking of multiple individual body parts, posture and frequency of movements. Activity parameters for horizontal traveled distance (mm) and vertical standing frequency (times) were used for analysis.

### 2.5 Body composition

Body composition of rats was measured at 5, 14, 21, 28 months of age by dual-energy x-ray absorptiometry (DEXA) (Lunar iDXA, GE Medical Systems, WI, USA) with small animal software package. DEXA scans were always performed under Tiletamine/Zolazepam (Zoletil 50, Virbac Lab, France) anesthesia after a 12-h fasting. Water was made accessible during the fasting period. Body composition data included bone percentage, muscle percentage, and fat percentage.

### 2.6 Tumor assessment

Tumor incidence is based on visibility of X-ray image and reconfirmed by physical appearance and body palpitation.

### 2.7 Statistical analysis

Longevity was assessed by Kaplan-Meier survival analysis. Independent t test was used to compare difference of all variables between the control and Rg1 groups for each single time point. Chi-square was used to compare the difference in tumor incidence between two groups during the entire treatment period. Two-way analysis of variance (ANOVA) with repeated measure was used to determine the main effect (age and supplement) and interactive effect (age and supplement) for all measures. Probability of type 1 error at 5% is considered significant. SPSS software (12.0 version, Chicago, IL, USA) was used for statistical analysis. Microsoft Excel was used for making figures. All results were expressed as mean ± standard error (SE).

## 3 Results and discussion

**Fig. 1:**
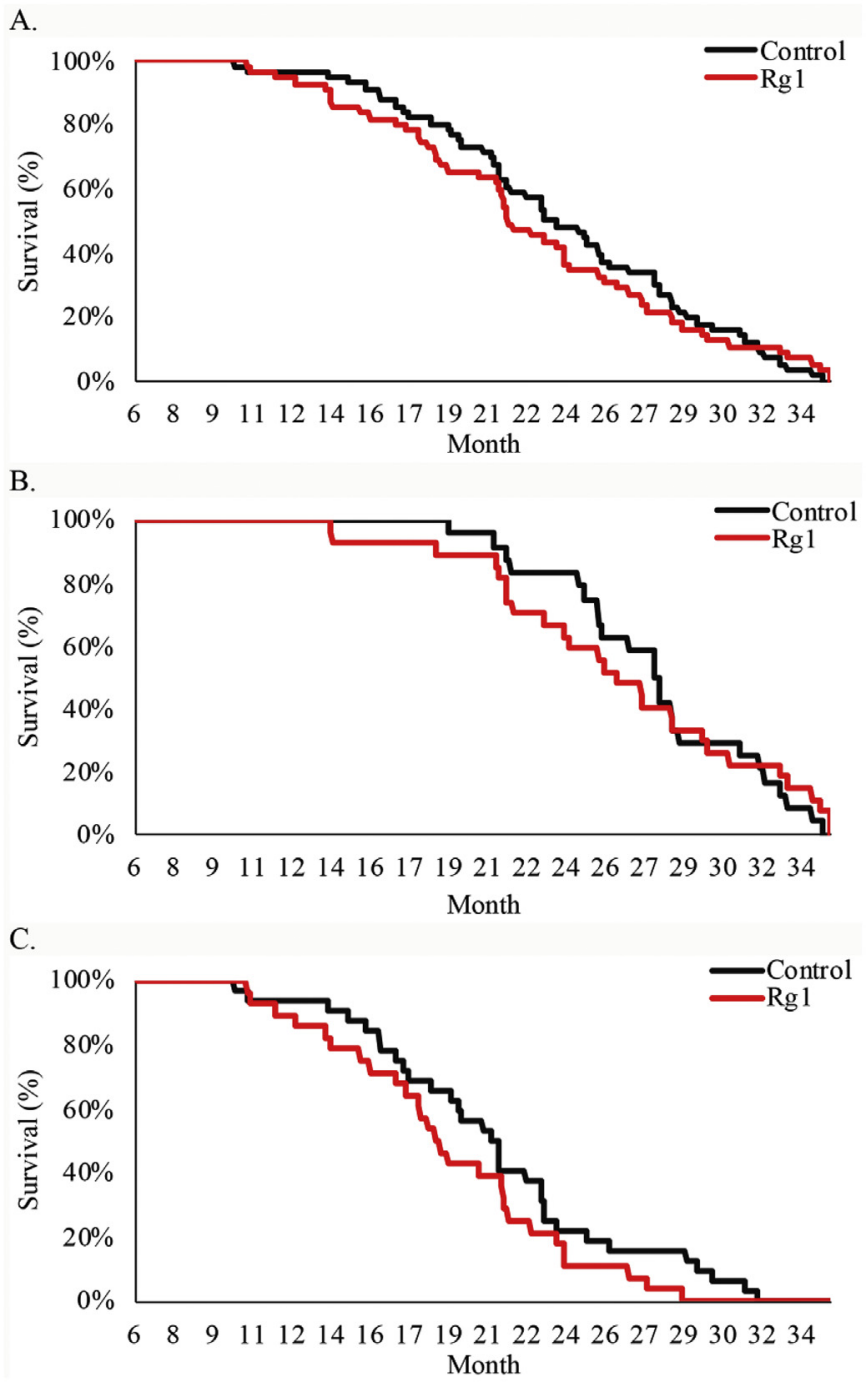
Effect of ginsenoside Rg1 supplementation on longevity. The survival time for both groups is presented for all (A) and separately for female (B) and male (C) rats. The control and Rg1 groups show similar result. Daily Rg1 dosage was 0.1 mg/kg.

**Fig. 2:**
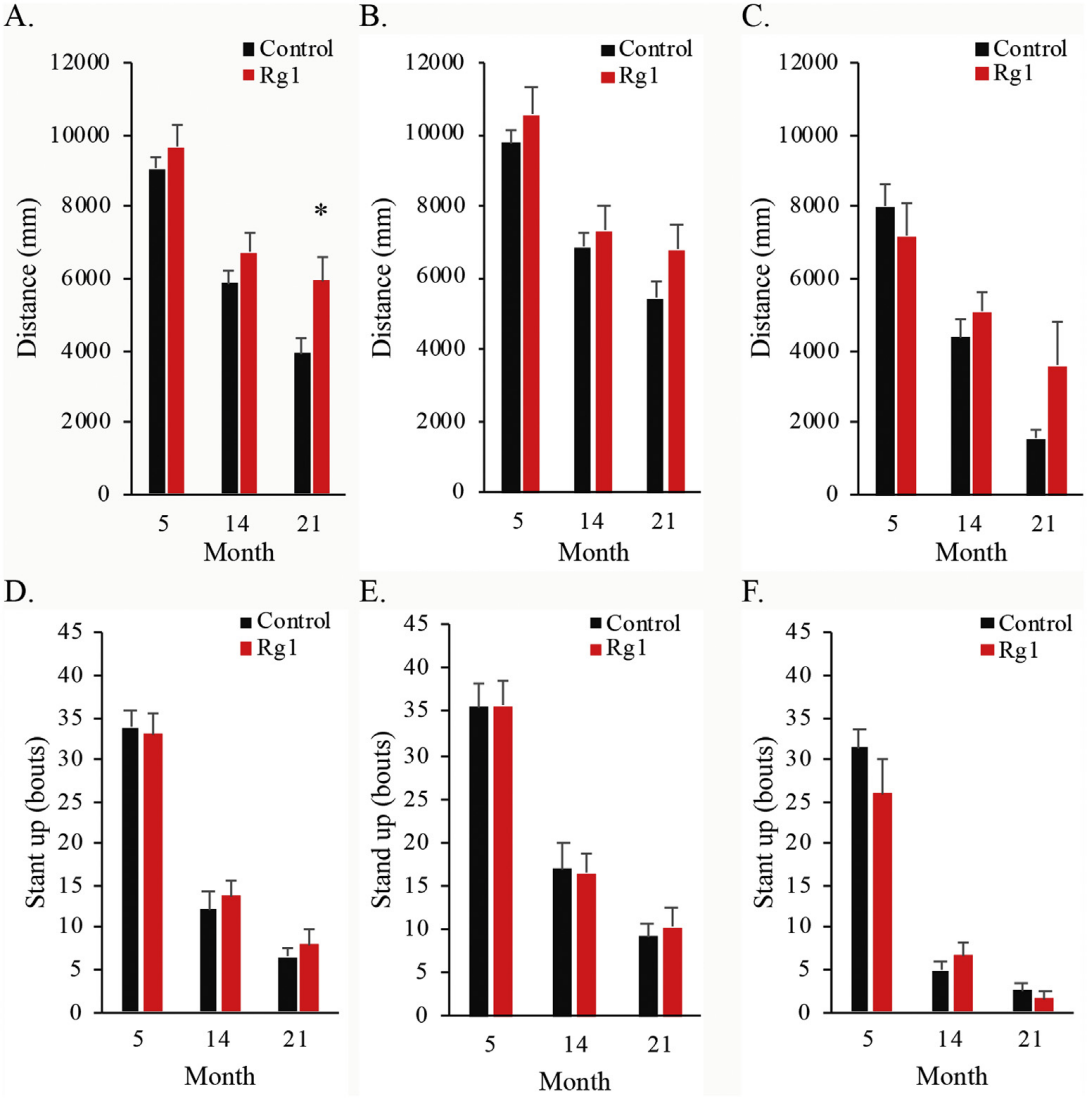
Effect of ginsenoside Rg1 supplementation on age-dependent declines in spontaneous physical activity during aging. ∗ Significantly difference against the control group, *p* < 0.05. Mean and standard error were calculated from survivors. Daily Rg1 dosage was 0.1 mg/kg.

Effects of lifelong ginsenoside Rg1 consumption on longevity and physical vitality have not been previously documented in mammals. In the study, longevity outcome was similar for the control and Rg1 groups (Fig. 1). Despite that male and female rats may have different pharmacokinetics and pharmacodynamics after oral Rg1 supplementation, similar results in longevity between both treatment groups were observed for male and female rats (Fig. 1B and C). The key finding of the study is a delayed age-dependent decline in spontaneous physical activity during late age in rats consuming ginsenoside Rg1 for the entire adulthood (Fig. 2). Significantly greater movement distance was observed in the Rg1 group at 21 months of age (*p* = 0.01), compared with rats in the control group (Fig. 2A). Both female rats (Fig. 2B) and male rats (Fig. 2C) show similar trend. The current data also provide novel evidence of neglectable toxicity of Rg1 for long-term use in adult rats based on Kaplan-Meier survival analysis. Most of the previous studies reporting Rg1 effect on spontaneous physical activity were conducted in young mice within a short period and were mostly under pathological conditions [7,9,10]. Taken together, the present study results suggest that lifelong consumption of Rg1 can improve physical vitality in naturally aging mammals but not longevity.

**Fig. 3:**
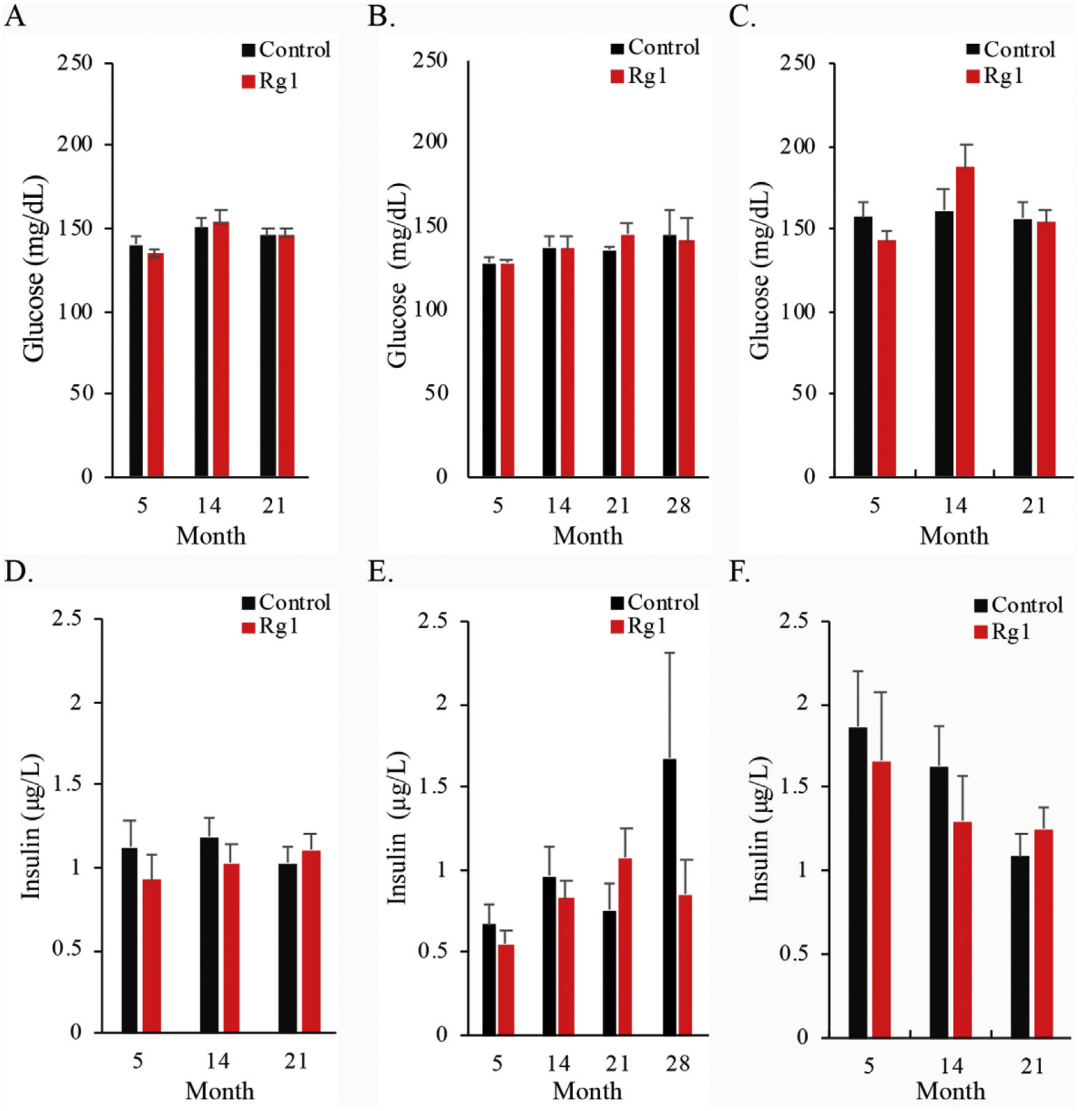
Effect of ginsenoside Rg1 supplementation on glucose and insulin levels during aging. No difference was found in circulating levels of glucose and insulin at 5, 14, 21, 28 months of age. Mean and standard error were calculated from survivors. Daily Rg1 dosage was 0.1 mg/kg.

**Fig. 4:**
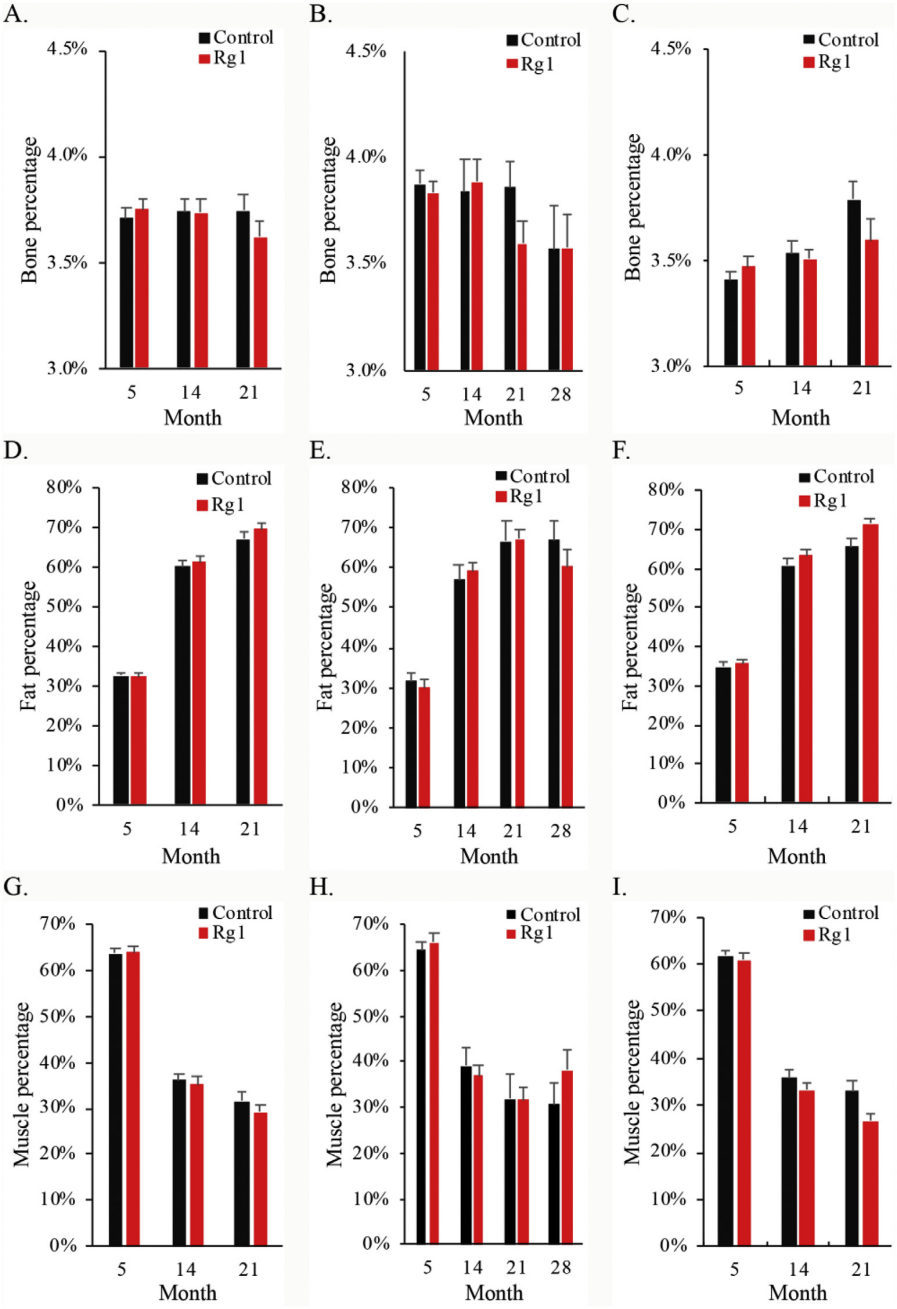
Effect of ginsenoside Rg1 supplementation on body composition during aging. While bone percentage remained unchanged (A, B, C), body fat percentage increased (D, E, F) and lean mass percentage decreased during aging (G, H, I). No difference between the control and Rg1 groups was observed for all body composition variables at 5, 14, 21, 28 months of age. Daily Rg1 dosage was 0.1 mg/kg.

Fig. 3 shows glucose and insulin trajectories of 21-month survivors after 5 months of age. Both metabolic measures in blood were similar for rats in the control and Rg1 groups across the entire observation period. Insulin resistance associated with overweight and obesity has been recognized as the common origin of metabolic problems and cancer in adults at higher age [13]. In this study, obesity (body fat accumulation) developed during the first 3 quarters of life, while muscle mass percentage decreases during the same period (Fig. 4). Effect of Rg1 supplementation on suppressing obesity has been previously reported [19]. However, we do not find significant effects of Rg1 on obesity and plasma insulin in the lifelong Rg1 supplementation study. The discrepancy may be associated with the fact that most of the previous Rg1 supplementation studies were conducted within a short period with much higher dosage in obese animal models which may be toxic to the animals [19].

**Fig. 5:**
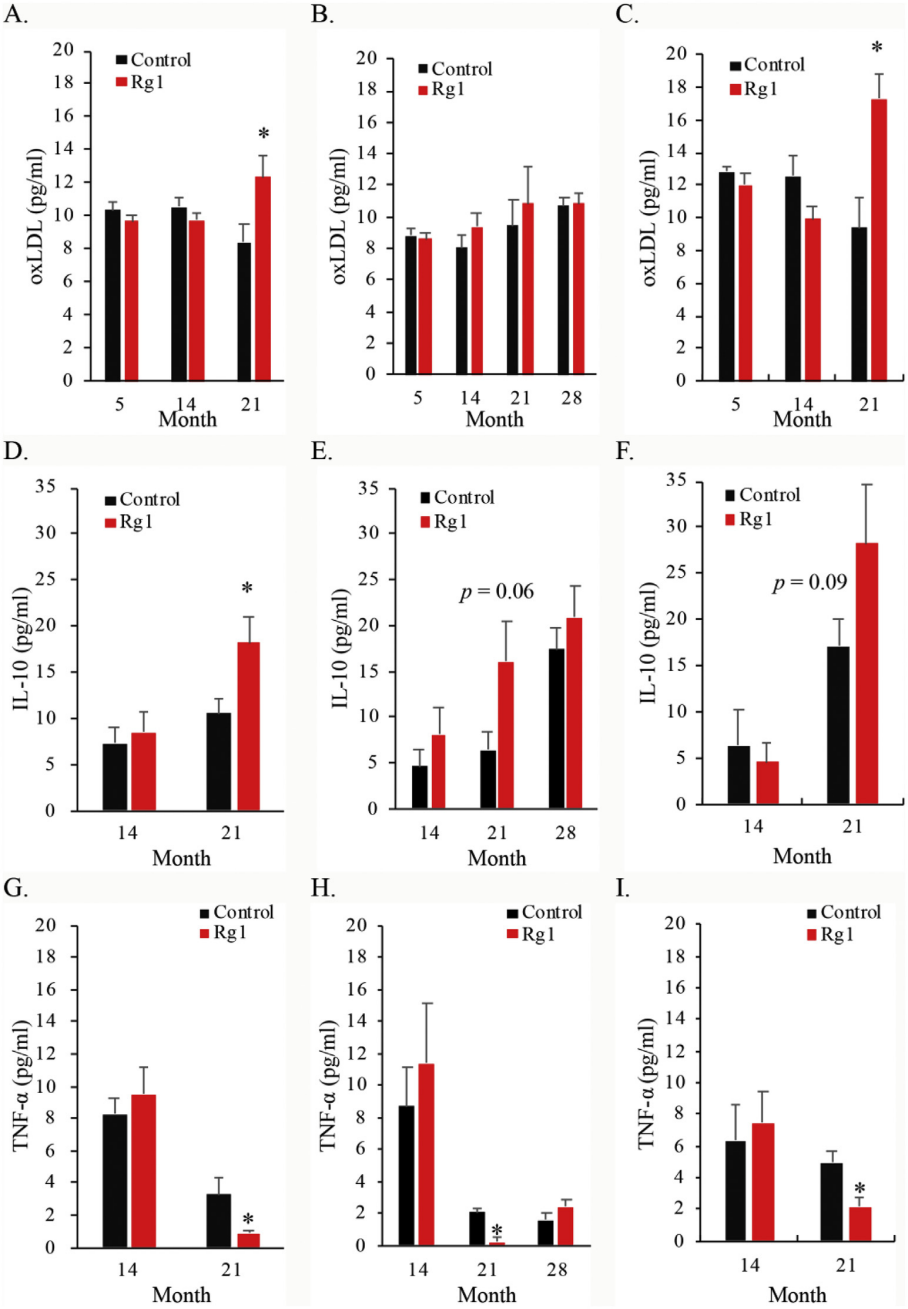
Effect of ginsenoside Rg1 supplementation on plasma inflammatory markers during aging. Rats consumed Rg1 show higher levels of oxidized LDL (A) and IL-10 (B) and lower levels of TNF-α (C) compared with the control group. ∗ Significantly difference against the control group, *p* < 0.05. Mean and standard error were calculated from survivors > 21 months (male) or 28 months (female) of age. Daily Rg1 dosage was 0.1 mg/kg.

Weight gain increases cell senescence [20] and cell death [21] in multicellular organisms, which elevates baseline inflammation levels [22]. Inflammation is an immune response which is essential to sustain the increasingly growing body with more cell population [23] by inducing phagocytosis to eliminate unhealthy senescent cells (with increased TNF-α) followed by triggering cell regeneration (with increased IL-10) [16]. Fig. 5 presents trajectory data for biomarkers involving inflammation, which includes plasma oxidized LDL, TNF-α, and IL-10 of survivors at 21 months of age. In support to a cross-sectional study comparing rats between 4 months and 18 months of age [14], we have further observed an increased IL-10 and a decreased TNF-α from 14 to 21 months of age, suggesting a shifting inflammatory balance from phagocytosis to regeneration [24]. This inflammatory shift may have been a compensatory modulation to sustain an increasingly larger cell population with more cell death in growing mammals. Here, we have observed an enhanced IL-10 elevation during aging in rats with Rg1 treatment, compared with that in the control group. This result suggests that Rg1 strengthens the compensatory mechanism in cell regeneration process by modulating the inflammatory balance during aging [16,25]. IL-10 is protective to the aging animals against the formation of foam cells induced by oxidized LDL [26]. Oxidized LDL in the rats consuming Rg1 drinks was significantly higher than the rats consuming control drinks at 21 months of age (*p* = 0.02), contributed mostly by male rats (*p* = 0.007). Survival time appears to be unrelated with oxidized LDL. IL-10 is a potent inhibitor for TNF-α expression in immune cells during inflammation [27,28]. Therefore, greater age-dependent increases in IL-10 observed in the study fit well with greater decreases in TNF-α in the Rg1-treated rats compared with the control group during advancing age.

**Fig. 6:**
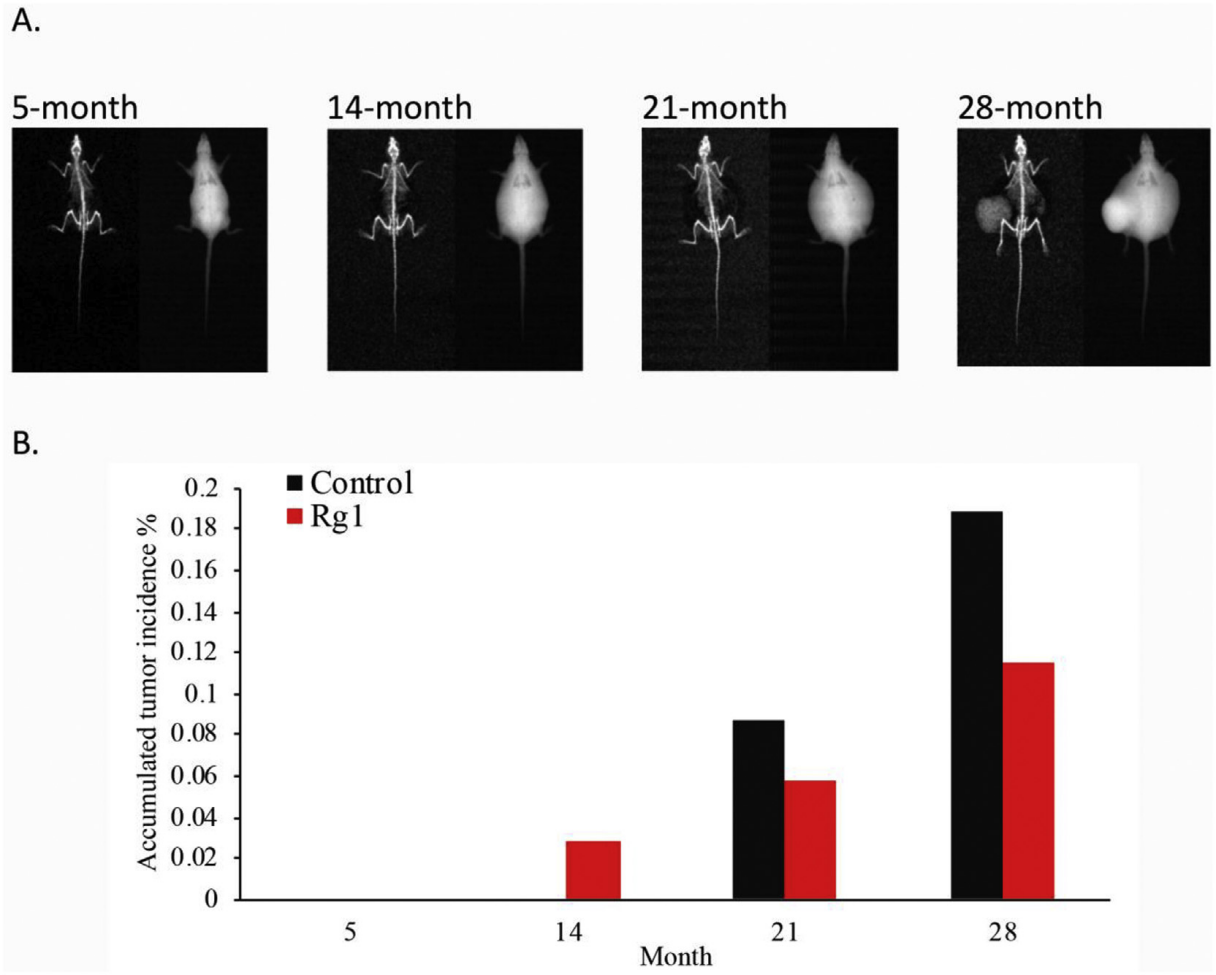
Effect of ginsenoside Rg1 supplementation on tumor incidence during aging. Representative images of visible tumors under X-ray from the same animal (A). No significant difference in tumor incidence between the control and Rg1 groups was observed (B). Daily Rg1 dosage was 0.1 mg/kg.

Weight gain is also the main contributor to tumorigenesis [29]. Tumor incidence increased with age and was similar for the control and Rg1 groups (Fig. 6). The absence of the Rg1 effect is probably associated the similar growth rate between both treatment groups. In the study, tumor incidence increased with age and reaching 19% in the control group and 12% in the Rg1 group of rats at 28 months of age. Difference in tumor incidence across the entire life between the control and Rg1 groups was not significant (*p* = 0.24).

The major limitation of the current work is that the single-dose study cannot preclude the possibility of a life-prolonging effect of Rg1 at different doses. Hormesis properties of ginsenosides have been reported in the past that antioxidant capacity increases only at low or moderate, not higher doses [30]. This result suggests that a multi-dose study for optimization is needed to conclude the effect of ginsenoside on longevity, tumorigenesis, and metabolic outcomes. Furthermore, Rg1 has been shown to stimulate senescent cell clearance associated with immune cell activation after exercise [8]. Effect of pre-exercise Rg1 supplementation on mortality and physical vitality during late life deserves more investigation. Another limitation of the study is that we could not distinguish whether the Rg1 action is mediated by its metabolites. Rg1 is quickly deglycosylated *in vivo* [31]. Therefore, further investigation is required to determine what metabolite of Rg1 mediates the improvement in physical vitality.

## 4 Conclusion

Physical vitality, reflected by decreased spontaneous physical activity, declines during late life. The results of the study demonstrated that this age-dependent decline can be effectively attenuated in rats consuming ginsenoside Rg1 during adulthood. This effect is associated with immunomodulatory effect of Rg1. However, the dose used in the study could not produce noticeable effects in longevity, body fat, tumors, and glycemic control.

## Author contributions

CCH, CYC, TXYL, JW, SS, and YWH performed experiments, analyzed data and developed figures. CCH and CYC analyzed data and developed figures. JW, SS, and YWH assisted with biological experiments. CCH and CYC wrote the draft. CHK and CYH designed the study and revised the manuscript. All other authors contributed to draft preparation.

## Declaration of competing interest

CHK and JFW are involved with US patents (10,806,764 B2) for an anti-aging method. This work was funded leading to a designed supplement Senactiv for Nuliv Science, USA.
